# Lack of partial renal response by 12 weeks after induction therapy predicts poor renal response and systemic damage accrual in lupus nephritis class III or IV

**DOI:** 10.1186/s13075-016-1202-z

**Published:** 2017-01-13

**Authors:** Hironari Hanaoka, Hidehiro Yamada, Tomofumi Kiyokawa, Harunobu Iida, Takeshi Suzuki, Yoshioki Yamasaki, Seido Ooka, Hiroko Nagafuchi, Takahiro Okazaki, Daisuke Ichikawa, Sayuri Shirai, Yugo Shibagaki, Junki Koike, Shoichi Ozaki

**Affiliations:** 1 Division of Rheumatology and Allergology, Department of Internal Medicine, St. Marianna University School of Medicine, Kanagawa, 216-8511 Japan; 2 Division of Nephrology and Hypertension, Department of Internal Medicine, St. Marianna University School of Medicine, Kanagawa, 216-8511 Japan; 3 Department of Pathology, St. Marianna University School of Medicine, Kanagawa, 216-8511 Japan

**Keywords:** Lupus nephritis, Induction therapy, Renal response, Damage accrual

## Abstract

**Background:**

Lupus nephritis class III or IV is associated with a poor prognosis for both patient and renal survival. Recommendations for the management of lupus nephritis have recently been established, and changing therapies is recommended for patients who do not respond adequately to induction therapy. However, it remains a major challenge to determine when to switch the treatment. In this study, we identified early prognostic factors capable of predicting poor renal outcome as well as overall damage accrual in patients with lupus nephritis class III or IV.

**Methods:**

Eighty patients with biopsy-proven lupus nephritis class III or IV were retrospectively recruited and divided into two groups: those with complete renal response (CR) or non-CR at 3 years after induction therapy. We investigated when clinical responses were obtained at each observational period from baseline to year 3. Clinical responses were divided into three groups: CR, partial renal response (PR), and non-PR. Furthermore, patients were assessed using the Systemic Lupus International Collaborating Clinics/American College of Rheumatology Damage Index (SDI) and cumulative dose of corticosteroid for 3 years.

**Results:**

Forty-four patients with CR and thirty-six with non-CR were enrolled. The cumulative CR rate was 85.0%. PR rates of patients with CR were significantly higher than those with non-CR from week 12 (*p* < 0.01). We identified the achievement of PR at 12 weeks as an independent predictor (OR 3.57, *p* = 0.03) by multivariate analysis. We next divided all patients into two groups according to PR achievement at week 12. The cumulative CR rate of the patients who achieved PR at week 12 was significantly higher than that of those who did not (96.5% vs 69.2%, *p* < 0.001). Furthermore, a significantly higher SDI and cumulative dose of corticosteroid were seen in the patients who did not achieve PR at week 12 than in those who did, regardless of their CR status, at year 3.

**Conclusions:**

Lack of PR at week 12 predicts a lower likelihood of achieving CR at 3 years and a higher SDI.

## Background

Lupus nephritis (LN) is a common manifestation of systemic lupus erythematosus (SLE) that contributes to significant morbidity and mortality [1]. Although the survival of patients with SLE has improved over the past several decades, the 10-year survival rate is still lower than that of age- and sex-matched healthy populations [2]. The most important cause of late mortality is cumulative organ damage [3]. Recently, the Joint European League Against Rheumatism and European Renal Association–European Dialysis and Transplant Association (EULAR/ERA-EDTA) published recommendations for LN management. They proposed that a partial renal response (PR) should be preferably achieved within 6 months after the initiation of treatment, and that treatment should be switched for patients without PR [4]. Furthermore, a treat-to-target approach developed to prevent flares and damage caused by SLE [5] suggests that patients should be properly monitored and therapy should be adjusted at reasonable time intervals. Given reports that early renal damage correlates with future damage accrual and is a predictor of a worse prognosis [6], an earlier decision to switch initial treatment for patients with poor renal response would be desirable in clinical settings. Although authors of a few reports have focused on the association between renal response and prognosis [7–11], there have been no reports on the early renal response as a predictive factor for overall damage accrual in patients with LN class III or IV. In the present study, we comprehensively analyzed patients with biopsy-proven LN class III or IV to determine early prognostic factors capable of predicting poor renal outcome as well as overall damage accrual.

## Methods

### Patients

We initially assessed the eligibility of all 358 consecutive Japanese patients with SLE who visited St. Marianna University Hospital from 2003 through 2010 and met the American College of Rheumatology classification criteria [12]. Among these, we identified patients diagnosed with class III or IV LN according to the International Society of Nephrology/Renal Pathology Society (ISN/RPS) classification [13] who had completed at least 3 years of observation. Of 88 patients with biopsy-proven LN, 82 had LN class III or IV. Two of these were lost to follow-up, and no patients died during the study period, leaving eighty patients for final enrollment. This study was approved by the ethics committee of St. Marianna University School of Medicine. Because this study was conducted with a retrospective cohort design without any samples taken besides those for clinical use, written informed consent was not acquired, in accordance with the guideline of Ministry of Health, Labour and Welfare of Japan. We retrospectively observed clinical characteristics, treatments, and clinical courses after initial induction therapy.

### Data collection

Clinical information was obtained from all patients at baseline and at 2, 4, 8, 12, 24, 48, 96, and 144 weeks (3 years) after initial induction therapy. The baseline clinical information was collected at the time of renal biopsy before initial induction therapy. Data gathered included demographic features, treatment regimens, and Systemic Lupus Erythematosus Disease Activity Index (SLEDAI) scores [14]. PR and complete renal response (CR) were defined on the basis of EULAR/ERA-EDTA recommendations for LN [4], with CR defined as a urine protein/creatinine ratio (UPCR) of 50 mg/mmol and normal or near-normal (within 10% of normal glomerular filtration rate [GFR] if previously abnormal) renal function and PR defined as a ≥50% reduction in proteinuria and normal or near-normal GFR. We substituted 0.5 g/g creatinine for UPCR 50 mg/mmol [4]. Renal relapse was defined as loss of CR status after achieving CR. Additionally, we used the Systemic Lupus International Collaborating Clinics/American College of Rheumatology Damage Index (SDI) to assess systemic damage accrual [15]. SDI was evaluated at baseline and at 6 months and 1, 2, and 3 years after induction therapy. We calculated the damage point presenting for at least 6 months [15].

### Prognostic factors for CR at 3 years

The primary endpoint was set as CR at 3 years after the initiation of induction therapy. We divided the patients into two groups based on whether they achieved this endpoint, and then we compared them regarding baseline demographic features, treatment regimens, and clinical course after the initiation of treatment. Further, we investigated when clinical responses were obtained during each observational period from baseline to year 3.

### Renal pathology

Patients underwent a renal biopsy before the initial induction therapy. In all cases, specimens taken for light microscopy were embedded in paraffin; sectioned; and stained with Masson’s trichrome, hematoxylin and eosin, periodic acid silver–methenamine, and periodic acid–Schiff. Frozen tissue was cut into 5-μm sections and incubated with fluoresceinated antisera to human immunoglobulins (Ig) IgG, IgA, and IgM; complement components C3, C4, and C1q; and fibrinogen. All patients were diagnosed according to the ISN/RPS classification [13] by light microscopy and immunofluorescence analysis. The activity index (AI) and the chronicity index (CI) developed by Austin et al. [16] were scored. Morphological features of the standard AI and CI were evaluated separately, namely endocapillary hypercellularity, polymorphonuclear leukocyte infiltration, karyorrhexis/fibrinoid necrosis, cellular crescents, hyaline deposits, interstitial inflammation, glomerular sclerosis, fibrous crescents, tubular atrophy, and interstitial fibrosis. We measured the percentage of these features in individual patients.

### Statistical analysis

Continuous values are shown as mean ± SD. Clinical characteristics between the two groups were compared using the nonparametric Mann-Whitney *U* test. Frequencies of clinicopathological characteristics were compared using the chi-square test. Cumulative CR rates were calculated using the Kaplan-Meier method, and differences between the two groups were tested with a log-rank test. To identify independent parameters that predict CR at 3 years after the initial therapy, we performed multivariate analysis using initial characteristics previously reported as predictors for good renal outcome [17], treatment regimens, and PR at 12 weeks. We selected SLEDAI and complement component CH50 levels as other covariates in multivariate analysis because they differed significantly between CR and non-CR patients at their baseline. Additionally, because therapeutic intervention may influence clinical response, particularly intravenous cyclophosphamide (IVCY) or mycophenolate mofetil (MMF) use [18, 19], we performed multiple regression analysis with baseline estimated glomerular filtration rate (eGFR), SLEDAI, CH50 level, IVCY use, MMF use, and achievement of PR at week 12 as dependent variables for CR at 3 years.

## Results

### Baseline clinicopathological characteristics and treatment regimens

We enrolled 80 patients and divided them into 2 groups according to their CR status at 3 years after induction therapy. At 3 years, 44 patients remained in CR and 36 did not. Demographic and clinical features of the patients at baseline are shown in Table 1. Among clinical features at baseline, patients with CR had significantly higher SLEDAI scores and lower CH50 levels (*p* < 0.01 and *p* = 0.02, respectively).

**Table 1:** Baseline clinical and renal pathological features of lupus nephritis patients with or without complete renal response at 3 years after induction therapy

Baseline characteristics	Complete renal response		*p* Value
	Achieved (*n* = 44)	Not achieved (*n* = 36)	
Female sex, *n* (%)	40 (90.9)	27 (75.0)	0.32
Age, years	39.7 ± 13.1	38.3 ± 11.5	0.43
BMI, kg/m^2^	22.1 ± 2.9	21.7 ± 3.1	0.31
Systolic blood pressure, mmHg	128.3 ± 16.7	130.0 ± 18.3	0.34
Diastolic blood pressure, mmHg	80.1 ± 13.2	79.9 ± 13.6	0.57
Disease duration, years	5.9 ± 8.0	7.7 ± 7.2	0.23
SLEDAI	16.3 ± 4.7	13.4 ± 4.9	<0.01
SDI	0.4 ± 0.6	0.5 ± 0.6	0.26
Proteinuria, g/g creatinine	2.6 ± 2.2	3.1 ± 1.8	0.14
eGFR, ml/minute	72.9 ± 27.4	77.6 ± 32.3	0.27
Anti-dsDNA antibody, IU/ml	212 ± 300	155 ± 259	0.26
Anticardiolipin antibody, IU/ml	23.5 ± 30.6	15.4 ± 25.6	0.12
Lupus anticoagulant-positive, *n* (%)	9 (20.5)	3 (8.3)	0.13
CH50, U/ml	16.1 ± 8.6	21.4 ± 12.4	0.02
Prednisolone, mg/day	45.9 ± 14.9	41.1 ± 14.1	0.07
Induction therapy, *n* (%)			
IVCY	25 (56.8)	15 (44.4)	0.34
MMF	8 (18.2)	3 (11.1)	0.23
Tacrolimus	7 (15.9)	6 (19.4)	0.96
PSL monotherapy	2 (4.5)	6 (16.7)	0.07
Others	2 (4.5)	6 (16.7)	0.07
Renal pathological findings			
ISN/RPS classification			
III (A) or III (A/C), *n* (%)	18 (40.9)	9 (25.0)	0.13
III (A) or III (A/C) + V, *n* (%)	4 (9.1)	6 (16.7)	0.34
IV (A) or IV (A/C), *n* (%)	14 (31.8)	15 (41.7)	0.41
IV (A) or IV (A/C) + V, *n* (%)	8 (18.2)	6 (16.7)	0.92
Endocapillary hypercellularity, %	41.2 ± 29.5	43.0 ± 33.1	0.27
Leukocyte infiltration, %	1.9 ± 5.5	1.4 ± 4.0	0.34
Subendothelial hyaline deposits, %	31.2 ± 32.7	30.7 ± 29.3	0.43
Fibrinoid necrosis/karyorrhexis, %	6.9 ± 12.4	15.3 ± 28.0	0.17
Cellular crescents, %	8.3 ± 7.3	10.6 ± 21.8	0.18
Interstitial inflammation, %	1.2 ± 4.8	2.1 ± 7.1	0.44
Glomerular sclerosis, %	3.7 ± 8.2	6.9 ± 9.1	0.25
Fibrous crescents, %	1.6 ± 3.1	1.1 ± 4.2	0.43
Tubular atrophy, %	3.8 ± 6.3	6.3 ± 6.1	0.14
Interstitial fibrosis, %	4.6 ± 7.2	7.1 ± 6.0	0.26
Activity index	5.1 ± 3.1	5.9 ± 4.1	0.21
Chronicity index	1.3 ± 0.2	1.8 ± 1.6	0.12

*Abbreviations: SLEDAI* Systemic Lupus Erythematosus Disease Activity Index, *SDI* Systemic Lupus International Collaborating Clinics/American College of Rheumatology Damage Index, *dsDNA* Double-stranded DNA, *IVCY* Intravenous cyclophosphamide, *MMF* Mycophenolate mofetil, *ISN/RPS* International Society of Nephrology/Renal Pathology Society, *BMI* Body mass index, *eGFR* Estimated glomerular filtration rate, *PSL* Prednisolone

All patients received glucocorticoid therapy at an initial dose of 1.0 mg equivalent prednisolone (PSL)/kg/day for 2–4 weeks. After initial therapy, PSL was tapered by 10% of the last dose or 10 mg, as determined by the attending physician. Eight patients were treated with PSL monotherapy, whereas others received immunosuppressive agents as induction therapy, including IVCY, MMF, or tacrolimus (TAC). The dose of IVCY ranged from 500 mg/2-week interval for six courses to 1000 mg/4-week interval for six courses. MMF was started at an initial dose of 0.5–1.0 g/day and gradually increased to 2.0 g/day. TAC dose (1.5–3.0 mg/day) was precisely adjusted to a trough value of serum concentrations. After six infusions of IVCY, patients were switched to azathioprine (AZA) at 100 mg/day while treatment with other immunosuppressants (ISs) was continued as maintenance therapy. Regarding initial treatment, PSL dose did not differ markedly between the two groups (*p* = 0.07), but a higher proportion of PSL monotherapy (*p* = 0.07) was observed in patients with non-CR. There were no remarkable differences between the two groups with regard to treatment regimens or in renal pathological analysis, including ISN/RPS classification, morphological features of LN, or AI and CI.

### Renal response at each visit and CR status at year 3

**Fig. 1:**
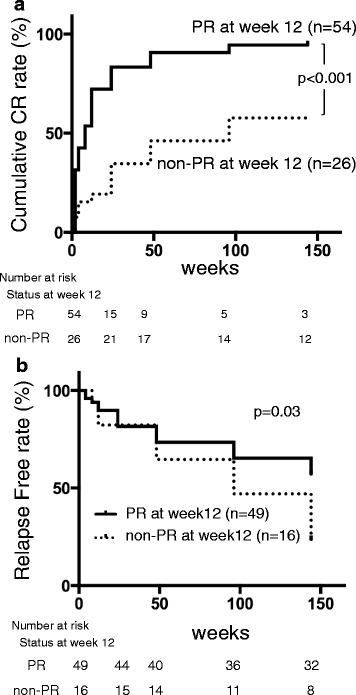
Cumulative CR rate and renal relapse-free rate for 3 years after induction therapy. **a** Cumulative CR rate is significantly higher in patients with PR at week 12 than in those with non-PR (HR 2.66, 95% confidence interval 2.13–5.47, *p* < 0.001). **b** Relapse-free rate is significantly higher in patients with PR at week 12 than in those with non-PR (HR 1.98, 95% confidence interval 1.10–5.35, *p* = 0.03). *CR* Complete renal response; *PR* Partial renal response

We next focused on renal response. Table 2 shows the percentage of patients achieving PR at each visit in the two groups. A significantly higher proportion of patients achieved PR in the CR than in the non-CR group from weeks 12 to 96 (*p* = 0.03, *p* < 0.01, *p* = 0.01, and *p* < 0.01, respectively). We further analyzed cumulative CR rates (Fig. 1). The cumulative CR rate for all patients was 85.0%. When we divided all patients into PR (*n* = 54) or non-PR (*n* = 26) at week 12, a significantly higher cumulative CR rate was seen in patients with PR at week 12 than with non-PR (96.5% vs 69.2%, *p* < 0.001). Although we conducted the same analysis of patients who achieved PR or non-PR at weeks 24, 48, and 96 separately, a significant difference was not detected in cumulative CR rates from the result for patients with PR or non-PR at week 12. Furthermore, a cutoff of 50.0% reduction of proteinuria from baseline at week 12 showed 79% sensitivity and 17% specificity (area under the ROC curve 0.73) for CR achievement at year 3. The analysis using 50% reduction of proteinuria at 3 months for SDI of 0 over 3 years showed 53% sensitivity and 79% specificity (area under the ROC curve 0.66). We next analyzed the relapse-free rate depending on PR achievement at week 12. We found 49 patients (90.7%) among those with PR at week 12 experienced CR during the 3 years, and 21 (42.9%) relapsed. Further, 16 patients (61.5%) in non-PR experienced CR, and 11 (68.5%) relapsed. A significantly higher relapse-free rate was found by the Kaplan-Meier method in those with PR at week 12 (*p* = 0.03) (Fig. 1b). Achieving PR at week 12 may indicate a higher likelihood of achieving CR and a lower likelihood of relapse for 3 years.

**Table 2:** Partial renal response at each visit and CR status at year 3

Observational period (week)	Patients who achieved PR, *n* (%)		*p* Value
	CR at year 3 (*n* = 44)	Non-CR at year 3 (*n* = 36)	
2	23 (52.5)	17 (47.2)	0.08
4	26 (59.1)	18 (50.0)	0.41
8	31 (70.4)	18 (50.0)	0.06
12	34 (77.3)	20 (55.5)	0.03
24	38 (86.4)	20 (55.5)	<0.01
48	40 (90.1)	25 (69.4)	0.01
96	40 (90.1)	25 (69.4)	0.01

*PR* Partial renal response, *CR* Complete renal response

### Identification of prognostic factors for CR at 3 years

We performed multiple regression analysis with baseline eGFR level, SLEDAI score, CH50 level, IVCY use, MMF use, and the achievement of PR at 12 weeks for the dependent variable CR at 3 years (Table 3). We statistically identified the achievement of PR at 12 weeks as an independent predictor (OR 3.57, *p* = 0.03). Although it has been well investigated that baseline renal function is an important predictor, we failed to show its significance. Because our patients had relatively mild renal dysfunction at baseline (75.0 ± 29.6 ml/minute/1.73 m^2^), we performed the analysis again by using the covariate of eGFR greater or less than 60 ml/minute/1.73 m^2^. The OR was 1.63 (95% confidence interval 0.64–4.33, *p* = 0.3).

**Table 3:** Multivariate analysis for predictors of patients with complete renal response at 3 years after induction therapy

Parameters	OR	95% confidence interval	*p* Value
PR at 12 weeks	3.57	1.16–12.1	0.03
eGFR, ml/minute/1.73 m^2^	1.00	0.98–1.02	0.62
SLEDAI	0.94	0.82–1.07	0.48
CH50, U/ml	1.03	0.97–1.09	0.21
MMF use	4.43	0.78–30.6	0.09
IVCY use	1.32	0.38–4.58	0.62

*Abbreviations: PR* Partial renal response, *SLEDAI* Systemic Lupus Erythematosus Disease Activity Index, *MMF* Mycophenolate mofetil, *IVCY* Intravenous cyclophosphamide, *eGFR* Estimated glomerular filtration rate

### Renal outcomes and damage accrual

**Fig. 2:**
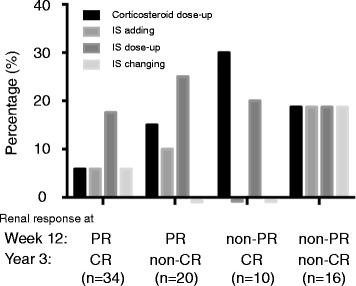
Intensification of therapy after induction therapy for 3 years. The percentage of patients treated aggressively, including corticosteroid dose-up, IS addition, IS dose-up, or IS change, depending on the renal response at week 12 and year 3, is shown. *IS* Immunosuppressant, *CR* Complete renal response, *PR* Partial renal response

We next classified all patients into four groups on the basis of PR status at week 12 and CR status at year 3. Their actual degree of improvement of proteinuria (percent change from baseline) is shown as follows (mean ± SD): patients with PR/CR (−90.6 ± 85.9%), PR/non-CR (−82.0 ± 79.3%), non-PR/CR (+16.0 ± 22.5%), and non-PR/non-CR (−17.2 ± 12.1%). Baseline clinical and renal pathological features of these groups are shown in Table 4. A significantly higher proportion of patients who experienced both PR at week 12 and CR at year 3 was female (*p* = 0.05) and had shorter duration (*p* = 0.09), but no other significant differences in clinical or renal pathological features at baseline were identified. Patients with PR/CR tended to have higher serological activity and SLEDAI at baseline than others. This may have influenced the physician’s therapeutic choice for aggressive therapy. More than 70% of patients with PR/CR and non-PR/CR received IVCY or MMF as an induction therapy, compared with less than 55% of those with non-PR/non-CR. Furthermore, more than 60% of patients with PR/CR received AZA or MMF as maintenance therapy, compared with 43.8% of those with non-PR/non-CR. Although the difference was not statistically significant, the small difference of the intensity of IS therapy may have resulted in our findings that patients with greater disease burden at baseline (higher serologic activity, SLEDAI, and LN class III or IV findings) belonged to the PR/CR group. We next evaluated the percentage of patients treated aggressively after the induction therapy, including corticosteroid dose-up, IS addition, IS dose-up, or IS change (Fig. 2). As a result, patients with PR/CR were not more aggressively treated after induction therapy than those in the other groups.

**Table 4:** Baseline clinical and renal pathological features of patients with systemic lupus erythematosus, depending on renal response at week 12 and year 3

	Renal response at week 12/year 3				
Baseline characteristics	PR/CR (*n* = 34)	PR/non-CR (*n* = 20)	Non-PR/CR (*n* = 10)	Non-PR/non-CR (*n* = 16)	*p* Value
Female sex, *n* (%)	33 (97.1)	15 (75.0)	7 (70.0)	12 (75.0)	0.05
Age, years	38.9 ± 12.8	38.8 ± 11.5	42.6 ± 14.5	37.8 ± 11.9	0.82
BMI, kg/m^2^	22.3 ± 3.1	21.6 ± 2.9	21.9 ± 2.9	21.9 ± 3.5	0.94
Systolic blood pressure, mmHg	128.8 ± 17.7	130.3 ± 15.4	126.5 ± 14.0	129.9 ± 22.1	0.91
Diastolic blood pressure, mmHg	80.8 ± 14.1	79.9 ± 13.3	77.4 ± 9.4	79.9 ± 14.6	0.86
Disease duration, years	4.6 ± 7.4	6.3 ± 6.5	10.8 ± 8.9	9.6 ± 7.9	0.09
SLEDAI	16.9 ± 4.5	14.1 ± 5.7	13.8 ± 4.9	12.6 ± 3.8	0.15
SDI	0.3 ± 0.6	0.5 ± 0.5	0.6 ± 0.7	0.6 ± 0.7	0.33
Proteinuria, g/g creatinine	2.4 ± 2.1	3.1 ± 1.9	2.9 ± 2.5	3.1 ± 1.7	0.47
eGFR, ml/minute/1.73 m^2^	73.5 ± 27.0	78.3 ± 32.2	70.9 ± 29.9	76.8 ± 33.4	0.84
Anti-dsDNA antibody, IU/ml	234.5 ± 332.1	106.3 ± 104.59	139.3 ± 150.1	212.9 ± 364.9	0.21
Anticardiolipin antibody, IU/ml	27.6 ± 14.1	19.1 ± 32.4	11.9 ± 7.7	10.5 ± 11.5	0.13
Lupus anticoagulant-positive, *n* (%)	8 (23.5)	2 (10.0)	1 (10.0)	1 (6.2)	0.34
CH50, U/ml	27.6 ± 14.1	19.1 ± 32.4	11.9 ± 7.7	10.5 ± 11.5	0.12
Prednisolone, mg/day	46.5 ± 14.0	41.5 ± 14.6	44.3 ± 18.6	40.7 ± 14.3	0.58
Induction therapy, *n* (%)					
IVCY	20 (58.8)	10 (50.0)	5 (50.0)	5 (33.3)	0.36
MMF	5 (14.7)	0 (0.0)	3 (30.0)	3 (18.8)	0.18
Tacrolimus	5 (14.7)	4 (20.0)	2 (20.0)	2 (12.5)	0.94
PSL monotherapy	2 (5.8)	4 (20.0)	0 (0.0)	2 (12.5)	0.15
Others	2 (5.8)	2 (10.0)	0 (0.0)	4 (25.0)	0.16
Maintenance therapy, *n* (%)					
Azathioprine	14 (41.2)	4 (20.0)	3 (30.0)	3 (18.8)	0.33
MMF	8 (23.5)	3 (15.0)	3 (30.0)	4 (25.0)	0.84
Tacrolimus	6 (17.6)	6 (30.0)	2 (20.0)	3 (18.8)	0.71
PSL monotherapy	4 (11.8)	4 (20.0)	1 (10.0)	1 (6.3)	0.66
Others	2 (5.9)	3 (15.0)	1 (10.0)	5 (31.3)	0.17
Renal pathological findings, *n* (%)					
ISN/RPS classification					
III (A) or III (A/C)	13 (38.2)	5 (27.9)	4 (40.0)	4 (25.0)	0.65
III (A) or III (A/C) + V	4 (11.8)	2 (10.0)	1 (10.0)	3 (18.8)	0.94
IV (A) or IV (A/C)	12 (35.3)	9 (45.0)	3 (30.0)	6 (37.5)	0.93
IV (A) or IV (A/C) + V	6 (17.6)	3 (15.0)	2 (20.0)	3 (18.8)	1.00
Endocapillary hypercellularity, %	31.2 ± 21.2	40.3 ± 13.1	41.3 ± 10.1	48.1 ± 8.2	0.64
Leukocyte infiltration, %	2.0 ± 3.5	1.1 ± 2.4	2.9 ± 1.3	2.2 ± 1.3	0.35
Subendothelial hyaline deposits, %	40.1 ± 10.1	29.1 ± 12.4	27.9 ± 12.7	38.1 ± 11.3	0.42
Fibrinoid necrosis/karyorrhexis, %	7.0 ± 10.1	6.0 ± 11.2	6.1 ± 10.4	5.9 ± 11.4	0.53
Cellular crescents, %	7.1 ± 7.2	8.1 ± 6.1	7.2 ± 7.4	8.8 ± 7.1	0.52
Interstitial inflammation, %	1.0 ± 4.8	1.2 ± 4.8	1.2 ± 4.8	1.2 ± 4.8	0.84
Glomerular sclerosis, %	3.9 ± 7.2	3.7 ± 8.1	3.2 ± 7.1	3.3 ± 6.2	0.71
Fibrous crescents, %	1.6 ± 3.0	1.5 ± 2.8	2.0 ± 1.1	1.7 ± 2.9	0.46
Tubular atrophy, %	5.2 ± 5.1	3.2 ± 5.1	3.1 ± 5.1	3.0 ± 6.1	0.27
Interstitial fibrosis, %	5.2 ± 6.2	4.1 ± 6.1	4.3 ± 6.3	4.0 ± 5.2	0.95
Activity index	5.3 ± 2.8	5.1 ± 2.9	5.4 ± 3.1	6.0 ± 2.1	0.27
Chronicity index	1.4 ± 0.1	1.3 ± 0.2	1.4 ± 0.1	1.8 ± 1.6	0.88

*Abbreviations: SLEDAI* Systemic Lupus Erythematosus Disease Activity Index, *SDI* Systemic Lupus International Collaborating Clinics/American College of Rheumatology Damage Index, *dsDNA* Double-stranded DNA, *IVCY* Intravenous cyclophosphamide, *MMF* Mycophenolate mofetil, *ISN/RPS* International Society of Nephrology/Renal Pathology Society, *BMI* Body mass index, *eGFR* Estimated glomerular filtration rate, *PSL* Prednisolone, *CR* Complete renal response, *PR* Partial renal response

**Fig. 3:**
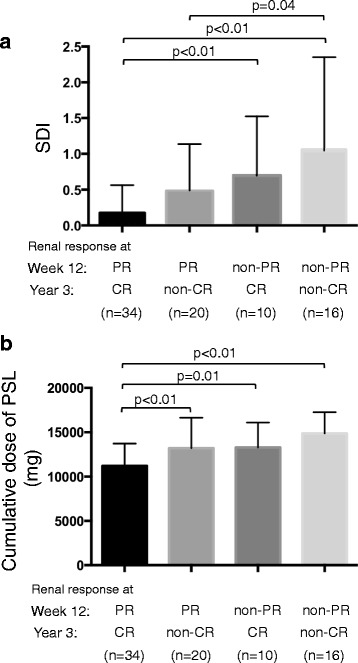
Systemic damage accrual and cumulative dose of prednisolone dose for 3 years after induction therapy. **a** Patients were divided into four groups on the basis of renal response at week 12 and year 3. A significantly lower SDI was seen in patients achieving both PR at week 12 and CR at year 3 than in patients who failed to achieve PR at week 12, regardless of CR achievement at year 3 (*p* < 0.01 and *p* < 0.01, respectively). **b** Compared with patients who achieved CR at year 3, the cumulative steroid dose was significantly lower in patients who achieved PR at week 12 than in those who did not (*p* = 0.01). *SDI* Systemic Lupus International Collaborating Clinics/American College of Rheumatology Damage Index, *CR* Complete renal response, *PR* Partial renal response, *PSL* Prednisolone

**Fig. 4:**
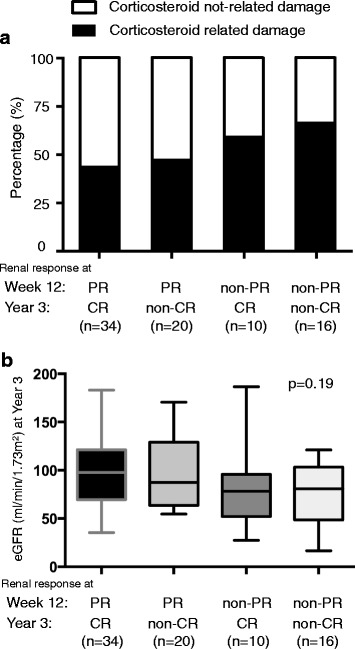
Component of SDI and renal damage at 3 years. **a** Percentage of corticosteroid-related or not corticosteroid-related damage of SDI in four groups. Corticosteroid-related damage was increased in patients with non-PR/non-CR. **b** The renal damage at year 3 in the four groups is shown. There is no significant difference in renal damage. *CR* Complete renal response, *PR* Partial renal response, *SDI* Systemic Lupus International Collaborating Clinics/American College of Rheumatology Damage Index, *eGFR* Estimated glomerular filtration rate

We analyzed SDI at year 3 among the four groups (Fig. 3a). Higher damage accrual was seen in patients who did not achieve both PR at week 12 and CR at year 3. Even when CR was obtained at year 3, patients who failed to achieve PR at week 12 had higher SDI values than those who did achieve PR at week 12 (*p* < 0.01). Collectively, SDI at year 3 depended primarily on PR status at week 12 and not on CR status at year 3. Among the three other groups, furthermore, the highest cumulative dose of corticosteroid for 3 years was seen in patients with both non-PR at week 12 and non-CR at year 3 (Fig. 3b). Compared with patients who achieved CR at year 3, those achieving PR at week 12 had a lower steroid dose than those who did not (*p* = 0.03). To investigate the impact of corticosteroid use on damage, we divided SDI into two categories (corticosteroid-related and not corticosteroid-related) according to the method of Gladman et al. [20]. Because the highest SDI was seen in the non-PR/non-CR group, we found the main damage was related to the corticosteroid (Fig. 4a). Because the cumulative PSL dose was increased (Fig. 3b), its damage also accumulated. Furthermore, we additionally investigated the renal damage at year 3 in the four groups (Fig. 4b). Although a slightly lower eGFR was found in the non-PR/CR group and the non-PR/non-CR group, there was no significant difference in renal damage. Early renal response and lower dose of corticosteroid were associated with lower systemic damage accrual.

## Discussion

In this study, we found that achievement of PR at week 12 was associated with low systemic damage at year 3, regardless of CR achievement. Further studies should address whether switching therapy is beneficial.

Most patients with SLE develop LN. LN impacts clinical outcomes in SLE, both directly by target organ damage and indirectly through complications of therapy, resulting in increased risks for renal failure, cardiovascular disease, and death [21]. The most important cause of late mortality in SLE is damage accrual, and SDI evaluation is a valuable means of predicting its prognosis [3]. Several factors contribute to the development of damage in patients with SLE [22]. To reduce systemic damage caused by disease activity, patients should be properly monitored, and therapy should be adjusted according to clinical status [5]. Because renal damage early in the disease course predicts worse prognosis, initial induction treatment should be promptly switched for patients who do not respond as expected. Among other factors, corticosteroid use has been established clearly to cause damage [23, 24]. Recently, Joo et al. demonstrated LN is associated with increased corticosteroid-associated damage compared with non-LN [24]. It is very important to aim for the lowest corticosteroid dose needed to control disease activity, especially for patients with LN [5]. In our study, patients who failed to achieve PR at week 12 tended to have higher SDI over 3 years. Its main damage was associated with corticosteroid use. Persistent LN activity leads these patients to be given a higher dose of corticosteroid. Because prevention of damage accrual should be a major therapeutic goal in SLE [5], PR at week 12 may be a clinically important target.

According to the EULAR/ERA-EDTA recommendations for LN management, PR should be achieved within 6 months after the start of induction treatment [4]. Our results, however, showed that more patients experienced PR by 6 months after induction treatment than by 3 months, and the cumulative CR rate at 3 years did not significantly differ between these two groups (*p* = 0.2). Further, only four patients (5.0% of all patients) who did not achieve PR at 3 months finally achieved PR at 6 months. Given that most patients who responded at 6 months after the start of induction treatment had already responded at 3 months, observation until 6 months might not be particularly beneficial.

Our results suggest that if patients do not achieve PR by week 12, they will be less likely to experience CR in the future and would accrue more renal damage, regardless of CR status at year 3. Our findings support a previous report by Rahman et al., who noted that early renal damage correlated with future damage accrual [6]. In an experimental lupus murine model, an earlier response to treatment was associated with an improved survival rate compared with a later response [25]. We speculate that the disease process may be less severe in fast responders and that these patients can sustain a good clinical course in the long term.

Authors of several previous reports investigated renal outcomes in patients with LN class III or IV by focusing on renal responses [7–11]. Illei et al. [7] and Mok et al. [8] evaluated mainly patients with LN treated with cyclophosphamide and reported that renal flares were common in patients with a sustained partial response compared with those with a complete response. Korbet et al. [10] investigated patients with LN treated with plasmapheresis and concluded that >50% reduction of proteinuria at 6 months predicted 15-year survival. Recently, Tamirou et al. [11] reported 10-year follow-up of the MAINTAIN Nephritis Trial and concluded that early decrease of proteinuria at 3 months showed a high positive predictive value but a low negative predictive value for good renal outcome. The MAINTAIN study comprised a European cohort, and a direct comparison with our data would not be appropriate; however, although the primary outcome was defined differently, a higher negative predictive value for early decrease of proteinuria was obtained in our results than in Tamirou et al.’s (62% vs 21%, respectively). Our results may suggest useful information for switching therapy in patients with lack of PR by 12 weeks. Furthermore, we also investigated the accrual of damage in all patients, depending on their early clinical response and future outcome. Because patients with LN should be closely managed to reduce damage accrual [5], our findings show the importance of early renal response in clinical settings. However, careful assessment would be needed regarding whether induction therapy should be modified in patients without PR at week 12, because renal response to cyclophosphamide sometimes takes longer (up to 12 months).

The present study is limited by its single-center, retrospective design and relatively short observational period. The differences in disease findings and intensity of therapy among the groups may have failed to show statistical significance, owing to the small sample size. The baseline clinical characteristics in the sample were quite variable, and not all the induction therapies were standard regimens. The IS doses were reduced in some patients because of safety concerns. Some patients refused the use of ISs. Therefore, these limitations may make our findings less convincing. The results of the study may not be applicable to the other population that received the standard regimens. Furthermore, the patients were all Japanese, and our data may not be generalizable to other ethnic groups. Renal response may differ depending on ethnic and racial background. Therefore, a multicenter, prospective study is required to confirm our findings. Our suggestion to switch induction therapy in patients without achieving PR at week 12 requires further analysis to determine whether switching treatment could reduce future damage in these patients.

## Conclusions

We found that achievement of PR at week 12 was associated with low systemic damage at year 3, regardless of CR achievement. Further studies should address whether switching therapy at 12 weeks if a PR is not reached results in an improved CR rate and a lower SDI score.

## Abbreviations

AI: Activity index
AZA: Azathioprine
BMI: Body mass index
CI: Chronicity index
CR: Complete renal response
dsDNA: Double-stranded DNA
eGFR: Estimated glomerular filtration rate
EULAR/ERA-EDTA: Joint European League Against Rheumatism and European Renal Association–European Dialysis and Transplant Association
GFR: Glomerular filtration rate
Ig: Immunoglobulin
IS: Immunosuppressant
ISN/RPS: International Society of Nephrology/Renal Pathology Society
IVCY: Intravenous cyclophosphamide
LN: Lupus nephritis
MMF: Mycophenolate mofetil
PR: Partial renal response
PSL: Prednisolone
SDI: Systemic Lupus International Collaborating Clinics/American College of Rheumatology Damage Index
SLE: Systemic lupus erythematosus
SLEDAI: Systemic Lupus Erythematosus Disease Activity Index
TAC: Tacrolimus
UPCR: Urine protein/creatinine ratio
